# Mechanisms modulating foam cell formation in the arterial intima: exploring new therapeutic opportunities in atherosclerosis

**DOI:** 10.3389/fcvm.2024.1381520

**Published:** 2024-06-17

**Authors:** M. T. La Chica Lhoëst, A. Martinez, L. Claudi, E. Garcia, A. Benitez-Amaro, A. Polishchuk, J. Piñero, D. Vilades, J. M. Guerra, F. Sanz, N. Rotllan, J. C. Escolà-Gil, V. Llorente-Cortés

**Affiliations:** ^1^ Department of Experimental Pathology, Institute of Biomedical Research of Barcelona (IIBB)-Spanish National Research Council (CSIC), Barcelona, Spain; ^2^ Department of Cardiovascular, Institut de Recerca Sant Pau (IR SANT PAU), Barcelona, Spain; ^3^ Research Programme on Biomedical Informatics (GRIB), Department of Experimental and Health Sciences (DCEXS), Hospital del Mar Medical Research Institute (IMIM), Universitat Pompeu Fabra (UPF), Barcelona, Spain; ^4^ Department of Cardiology, Hospital de la Santa Creu I Sant Pau, Biomedical Research Institute Sant Pau (IIB-SANTPAU), Universitat Autonoma de Barcelona, Barcelona, Spain; ^5^ Department of Cardiovascular, CIBERCV, Institute of Health Carlos III, Madrid, Spain; ^6^ Department of Cardiovascular, CIBERDEM, Institute of Health Carlos III, Madrid, Spain

**Keywords:** apolipoproteins (ApoB100, apoA1, apoC1, apoJ, apoE), foam-like SMC, atherosclerosis, cardiovascular diseases, peptidomimetics, lipoproteins, transcription factors, reverse cholesterol transport

## Abstract

In recent years, the role of macrophages as the primary cell type contributing to foam cell formation and atheroma plaque development has been widely acknowledged. However, it has been long recognized that diffuse intimal thickening (DIM), which precedes the formation of early fatty streaks in humans, primarily consists of lipid-loaded smooth muscle cells (SMCs) and their secreted proteoglycans. Recent studies have further supported the notion that SMCs constitute the majority of foam cells in advanced atherosclerotic plaques. Given that SMCs are a major component of the vascular wall, they serve as a significant source of microvesicles and exosomes, which have the potential to regulate the physiology of other vascular cells. Notably, more than half of the foam cells present in atherosclerotic lesions are of SMC origin. In this review, we describe several mechanisms underlying the formation of intimal foam-like cells in atherosclerotic plaques. Based on these mechanisms, we discuss novel therapeutic approaches that have been developed to regulate the generation of intimal foam-like cells. These innovative strategies hold promise for improving the management of atherosclerosis in the near future.

## 1 Introduction

Cardiovascular diseases (CVDs) are one of the leading causes of death worldwide, accounting for 32% of all global deaths (1). Among them, atherosclerosis is predominant, leading to tissue ischemia and events such as strokes and heart attacks, depending on the vascular territory affected. Atherosclerosis is characterized by the progressive accumulation of cholesterol and fibromatous material within the tunica intima of arterial walls, resulting in the formation of an atheroma plaque, which can obstruct blood flow (2). Human atherosclerosis is initiated by the retention of low-density lipoproteins (LDLs), positively charged, by negatively charged glucosaminoglycans (GAGs) of proteoglycans (PGs) that structure the extracellular matrix (ECM) in the arterial intima (3). In this context, Camejo et al, pioneers within the field, identified the specific segments of ApoB100 involved in the interaction of LDL with arterial PGs (4), and show that human proteoglycans have high affinity for fused LDL particles (5) and cytokines and growth factors (6).

Previous evidence suggests that diffuse intimal thickening (DIM), preceding early fatty streaks in humans, mainly consists of lipid-loaded SMC and their secreted proteoglycans (7). However, it was widely accepted for several years that macrophages were the major cell type forming foam cells in the arterial intima and the main contributors to atheroma plaque formation (8). Recent studies utilizing lineage-tracing in mice have provided evidence that SMCs are not only the initial foam cells in the lesion but also comprise the majority of foam cells in advanced atherosclerotic plaques (9, 10). *In vitro* studies have demonstrated that human coronary SMCs readily uptake aggregated LDL (agLDL), leading to foam cell formation (11–13). Upon cholesterol loading, SMCs downregulate contractility markers such as alpha-actin, alpha-tropomyosin, myosin heavy chain, and calponin H1 while upregulating macrophage-related markers CD68, Mac-2, and ABCA1 (14)*.* Cell lineage tracing technology and other cutting-edge methods, such as single-cell RNA sequencing, have enabled the definition of SMC reprogramming towards various phenotypes, including osteochondrogenic, myofibroblastic, and mesenchymal stem cell-like phenotypes, in addition to the macrophage-like phenotype (15, 16). Depending on the activation of specific transcription factors, SMCs switch to dedifferentiated transitional states that can be pathological (such as macrophage-like) (17) or beneficial (such as protective fibromyocytes forming the fibrous cap) (18). Currently, the contribution of SMCs to atherosclerosis is undoubtedly greater than previously thought, as genome-wide association studies (GWAS) have highlighted the pivotal role of SMC-specific gene expression in predicting coronary artery disease risk in humans (19, 20).

In this review, we will provide an overview of the main factors modulating the formation of foam-like cells, particularly foam-like SMCs, and the mechanisms involved in their contribution to the progression of atherosclerosis. Additionally, we will review innovative proposals to reduce the onset and/or progression of atherosclerosis by modulating foam-like cell generation. These new therapeutic approaches could be essential for improving coronary artery disease management and preventing coronary acute events in the near future.

## 2 Unique crucial mechanisms for SMC transdifferentiation into foam cell-like

### 2.1 Transcriptional regulation of SMC phenotype

In response to microenvironmental factors, SMCs undergo transcriptional reprogramming, often referred to as phenotypic modulation, and migrate from the media to the inner membrane. This process involves the loss of classic contractile function and an increase in proliferative and synthetic abilities (9, 21). The role of phenotypically modulated SMCs in atherosclerosis can be both beneficial and detrimental. On one hand, SMCs contribute to the thickness of the fibrous cap through ECM synthesis, promoting plaque stability (18). On the other hand, a plethora of new SMC phenotypes including foam-cell like phenotype coexist in the arterial intima and contribute to the formation of the necrotic core and the progression of atherosclerosis (17, 22). Statins, the most prescribed hypolipemiants in the world, reduce the number of SMC in the arterial intima of hyperlipidemic rabbits (23) and reduce calcification via restoration of the Gas6-Axl pathway in vascular SMC (24). However, treatments targeting exclusively SMC phenotypic modulation and/or transdifferentiation are currently lacking. Interventions aimed at regulating the SMC phenotype have the potential to alter the progression of atherosclerotic plaques, as demonstrated in murine experimental models lacking specific transcription factors (17, 25–28).

A crucial transcription factor for lipid-induced transdifferentiation of SMCs into macrophage-like cells is the zinc finger transcription factor Krüppel-like factor 4 (KLF4). Lineage-tracing studies in ApoE−/− mice have shown that SMC-specific conditional deficiency in KLF4 (SMC YFP+/+ Klf4Δ/Δ Apoe−/−) as well as loss of one Klf4 allele globally in all cell types (ERT-Cre + Klf4Δ/WT Apoe−/−) decreased lesion size, apoptosis, and proliferation activities in the lesion. These models also exhibited increased plaque stability, including an increase in the thickness of the fibrous cap, indicating that KLF4 deficiency has mitigating effects on plaque development and progression. This suggests KLF4 in SMC as a potential therapeutic target for inducing smaller and more stable lesions (25). Furthermore, the KLF4 reduction promoted by angiopoietin-like 4 (ANGPTL4)-transforming necrosis factor alpha (TNFα)-NADPH oxidase 1 (NOX1) axis plays a role in the protective effect of the anti-inflammatory ANGPTL4 protein in atherosclerosis. Among other anti-atherogenic effects such as reduction of cardiac and vascular inflammation, ANGPTL4 reduces the phenotypic transition of SMCs into macrophage-like cells in the ApoE−/− mouse model of atherosclerosis (29).

Although there is no doubt that KLF4 plays a critical role in regulating phenotypic transitions of arterial smooth muscle cells (SMCs) and overall atherosclerotic lesion pathogenesis, additional studies in the SMC YFP+/+ Klf4Δ/Δ Apoe−/Δ murine model have evidenced a cardioprotective role of KLF4. This role was observed in reducing peripheral vascular resistance, dilated heart, and increased vascular leakage, all of which are changes related to the dysregulation of platelet-derived growth factor (PDGF) and fibroblast growth factor (FGF) signaling in association with KLF deficiency in SMCs. These results have implications regarding the essential role of KLF4 in the maintenance of microvascular SMC structure and function, as well as in the engraftment of supporting arteries (30). In line with the potential protective role of KLFs in the microvasculature, extracellular vesicles enriched in miR-143/145 derived from KLF2-expressing endothelial cells have been shown to reduce atherosclerotic lesion formation in the aorta of ApoE(−/−) mice (31). Additionally, *in vitro* studies in endothelial-SMC co-cultures have demonstrated that miR-143/145-enriched extracellular vesicles from endothelial cells target crucial atherosclerotic genes in SMCs such as ELK1 (32), KLF4 (33), CAMK2d (32), and SSH2 (33). The authors proposed that the modulation of atheroprotective miRNA- and extracellular-vesicle-mediated mechanisms could be a promising strategy to manage atherosclerosis. Controversially, the inhibition of miR-143/145 in western diet-fed LDLR KO mice (Ldlr−/−-miR-143/145−/−) has been shown to reduce atherosclerosis progression through switching from a contractile/non-proliferative state to a migratory/proliferative SMC state and hepatic ABCA1 expression (34). Consistent with these results, previous studies have evidenced increased levels of miR-145 in human carotid atherosclerotic plaques from symptomatic patients (35). These controversial results about the beneficial or pathological effect of miR-145 require the reevaluation of miR-145 effects in the context of dyslipidemia and cardiovascular-related diseases before assessing its potential clinical utility.

Another transcription factor crucially involved in the phenotypic transition of SMCs into CD68 + macrophage-like cells is BCLAF1 (BCL2-associated transcription factor 1). Integrating data from *in vitro* and *in vivo* experimental models and from humans, it has been demonstrated that BCLAF1 is implicated in the lipid-induced transdifferentiation of SMCs into macrophage-like cells, as it was shown to control lipid uptake in SMC via regulation of *CD36* and *CD68* scavenger receptors (36). Immunopositivity of BCLAF1 was found to colocalize with BCL2-positive areas but not with apoptotic areas of the necrotic core in the atherosclerotic plaques of SMC lineage-tracing atherosclerotic mice. Furthermore, in humans, downregulation of BCLAF1 was associated with plaque vulnerability parameters and higher cardiovascular risk in patients with carotid atherosclerosis (36). The transcriptional modulation of SMC phenotype is influenced by various environmental cues such as lipids, inflammatory cytokines, and altered cell-cell interactions. Indeed, the heterogeneity of SMCs in the arterial intima may reflect, at least in part, the dynamic nature of SMC responses to the atherosclerotic microenvironment. In the following sections, we will review the reported effects of some of these environmental cues on SMC phenotype.

### 2.2 Regulation of SMC phenotype by retinoic acid

Retinoic acid (RA) is a metabolite of vitamin A and a morphogen that governs cell differentiation and embryonic patterning in early developmental stages. Specifically, all-trans retinoic acid (tRA) inhibits proliferation while increasing migration and tissue-type plasminogen activator activity in two phenotypically distinct rat SMC populations, cultured respectively from the normal aortic media and from the intimal thickening (IT) after endothelial injury (37). These authors also demonstrated that retinoids reduce IT induced by balloon endothelium injury in a rat experimental model. Further studies explored the effects of tRA, either alone or in combination with basic fibroblast growth factor (bFGF), on the expression of plasminogen activator inhibitor-1 (PAI-1) in cultured vascular SMCs. PAI-1 expression in SMCs regulates the extracellular matrix composition, which, in turn, influences SMC migration, among other relevant aspects for atherosclerosis. tRA alone or in combination with bFGF upregulates PAI-1 synthesis by rabbit aortic SMCs, consequently enhancing SMC migration (38).

Recent studies have explored how the stiffness of the extracellular environment influences cellular processes and have pointed to discoidin receptor 1 (DDR1) as a crucial perceptive receptor whose phosphorylation, aggregation, and oligomerization depend on ECM stiffness. DDR1 activation was observed in collagen (Col)-coated gels and in calcified atherosclerotic plaques, resulting in changes in SMC genes modulated through extracellular signal-regulated kinases (ERKs) and p53 pathways, and related to inflammation and ECM homeostasis (39). These results have implications for the development of innovative ECM-based therapies useful for improving the management of atherosclerosis, as well as other prevalent pathologies such as cancer and fibrosis.

### 2.3 Effect of growth factors and inflammatory mediators on SMC phenotype

Besides interactions with neighboring cells and the ECM, inflammatory mediators released in response to injury or disease are key drivers of SMC phenotypic modulation (40). Basic fibroblast growth factor (bFGF) and heparin-binding epidermal growth factor-like growth factor (HB-EGF) induce morphological changes, disrupt SMC monolayers, and promote proliferation and dedifferentiation of SMCs (41). Interestingly, activating TGF-β signaling by inhibiting bFGF signaling prevents the switch from a contractile to a proliferative phenotype in SMCs and significantly reduces atherosclerotic plaque size in male mice fed a Western diet (42). These beneficial effects were linked to features of plaque stability sch as increased fibrous cap and reduced necrotic core in this experimental model. Together, they suggest a potential therapeutic effect of TGF-β/bFGF axis for managing coronary artery disease. Additionally, TGF-beta and Notch signaling pathways cooperatively promote a SMC contractile phenotype by co-regulating Smad activity at SMC promoters (43). Interferon-γ (IFN-γ), primarily secreted by macrophages and T cells, plays a crucial role in immune function against pathogens. IFN-γ stimulates SMC migration to the arterial intima, increases the number of intimal SMC-derived foam cells, promotes oxidative stress, and contributes to plaque formation and rupture (44). Conversely, IFN-γ has also been reported to reduce LDL uptake by macrophages, suggesting an anti-atherogenic role during foam cell formation (45). IL-1β and TNF-α enhance macrophage foam cell formation, accelerate the enlargement of unstable lipid-rich atherosclerotic plaques, and decrease lipid efflux, thereby promoting macrophage foam cell formation (46).

### 2.4 Effect of reactive oxygen Species (ROS) on SMC phenotype

Reactive oxygen species (ROS) production is increased in the vessel walls in pathological conditions such as hypertension, diabetes, smoking, and dyslipidemia (47). ROS induces proliferation (48, 49), and phenotypic switching (50) of SMC, through different mechanisms. ROS upregulates the production of growth factors and hypertrophic hormones including phenylephrine, thrombin, vascular epidermal growth factor (VEGF), basic fibroblast growth factor (bFGF), PDGF, insulin-like growth factor-I (IGF-I), and angiotensin II by vascular cells (48, 51–53). In addition, ROS induces SMC migration through the activation of matrix metalloproteinase (MMP) (54), responsible for matrix degradation and reorganization, facilitating SMC migration and proliferation (55, 56).

NADPH oxidase (NOX) enzymes present in the vascular wall are major contributors to ROS production and signaling in the vasculature (57). Multiple studies have evidenced an association between hyperactivation of NADPH oxidases and vascular pathologies, including atherosclerosis and restenosis (58). NOX1 is the NADPH oxidase isoform mainly expressed by SMC and one of the primary enzymes regulating SMC activation (59). NOX1-induced ROS production upregulates vascular inflammation in early atherosclerotic lesions of ApoE−/− mice (60). In line, NOX1/NOX4 pharmacological inhibition (61) and Nox1 deficiency (62) significantly attenuate vascular ROS levels and atherosclerosis burden in ApoE−/− mice.

NOX activator 1 (NOXA1), a critical functional homolog of p67phox for NADPH oxidase activation in mouse VSMC (63), is upregulated by TNF-α and angiotensin 2 in SMC, causing SMC activation (64). NOXA1-dependent NOX1 activation promotes SMC proliferation and migration and KLF4-mediated transition to macrophage-like cells, plaque inflammation, and expansion (65). These results support a critical role of NOX1 in the pathogenesis and progression of atherosclerosis. NADPH oxidase 4 (Nox4) is a constitutively active ROS-producing enzyme that is highly expressed in the vascular endothelium. The NOX4 isoform constitutively generates hydrogen peroxide (H2O2) in normal non-disease conditions. H2O2 production is tightly controlled in terms of levels and subcellular locations to mediate various physiological processes in vascular cells including the maintenance of the contractile SMC phenotype (66). Under hyperglycemic/diabetic conditions, NOX4 overexpression in endothelium is associated with atherosclerosis reduction in ApoE−/− mice (67). Di Marco et al. showed that NOX4 counteracts hyperglycemia-induced SMC proliferation and fibrosis and maintains a differentiated SMC phenotype in ApoE−/− mice (68). In these diabetic ApoE−/− mice, genetic deletion of NOX4 increases PDGF, osteopontin (OPN), and ECM protein fibronectin in aortic SMC concomitantly with elevated NOX1-associated ROS levels. Additional studies have consistently shown that genetic deletion of NOX4 increases atherosclerosis in ApoE−/− mice (69).

## 3 New factors besides hypercholesterolemia contribute to vascular foam cell formation, offering avenues for innovative therapies

### 3.1 LDL atherogenicity and new peptidomimetics keeping LDL integrity

Previous studies have demonstrated that aggregated LDL is a potent stimulus for foam cell formation from human coronary SMCs (11–13). Early research indicated that when LDL aggregates were removed from LDL preparations, LDL did not induce intracellular cholesteryl ester accumulation in SMC (70–72). Currently, individual susceptibility to LDL aggregation has been directly linked to future cardiovascular events (73), underscoring the crucial relevance of the interaction between aggregated LDL and SMC for atherosclerosis progression.

The susceptibility of LDL particles to aggregate is strongly associated with the particle's lipid composition, as evidenced by samples from a nested case–control study designed from the Finnish Corogene study (73). In this study, human LDL particles prone to aggregation were those enriched in sphingomyelins (SMs) and ceramides (Cer), with fewer phosphatidylcholine (PC) and triglyceride (TG) species compared to aggregation-resistant LDL particles. Additionally, dietary factors and/or medication can modify susceptibility to LDL aggregation. Increasing dietary intake of vegetable oils and plant stanol-enriched spreads, for example, reduces the propensity for LDL aggregation (74). Moreover, proprotein convertase subtilisin/kexin type 9 (PCSK9) inhibitors have been shown to reduce LDL aggregation by altering the lipid composition of LDL particles (75). Therefore, dietary factors that modulate LDL lipid composition and susceptibility to aggregation appear to be key determinants for foam SMC generation. A crucial factor in the diet that determines individual susceptibility to LDL aggregation is the type of fat (76). Camelina sativa oil (CSO), a source of alpha-linolenic acid (ALA), decreases the relative concentrations of saturated and monounsaturated cholesteryl ester species and increases polyunsaturated TGs in LDL (77). Additionally, dietary n-3 polyunsaturated fatty acids (PUFAs) are incorporated into the lipid species of LDL particles, increasing the degree of unsaturation of phospholipids and neutral lipids in the LDLs, thereby altering the tendency of LDL to aggregate. As mentioned above, LDL aggregation seems to be inversely associated with specific unsaturated species of PC and directly associated with those of SM. Therefore, depending on which specific species of phospholipids incorporate the unsaturation and the degree of this unsaturation in the LDL particle, the susceptibility may change (73, 75).

Currently, apolipoprotein-based peptides and their mimetics with the capacity to improve lipid profile and/or lipoprotein functionality have emerged as potential therapeutic tools for atherosclerosis (78). These peptides are usually designed as short amino acid chains based on functional domains of ApoA1 (79) 79 and ApoJ (80, 81) that due to their amphipathic nature have efficacy to rearrange lipids, in particular phospholipids, thereby conferring stability to LDL. Unlike ApoB-based peptides that interact with phospholipids, LRP1-based peptides target a particular sequence of ApoB100, stabilizing its conformation and efficiently preventing LDL aggregation (by more than 85%) (82, 83). These peptidomimetics emerge as potential tools to maintain LDL integrity in the arterial intima.

### 3.2 Proteoglycans and proteoglycan-based therapies

SMC switching from a contractile to a synthetic phenotype in the arterial intima promotes an increase in the proteoglycan production (84). Since proteoglycans contribute to LDL retention and modification (3, 7), intimal PGs and retained LDL create a vicious cycle driving foam SMC formation. Therefore, active immunization against GAG side chain of PGs has emerged as a promising strategy to target atherosclerosis due to the capacity of GAGs to retain and modify lipoproteins in the arterial intima (85).

Treatment of VSMCs with angiotensin II, the main effector of hypertension, promotes the production of elongated GAG chains, which are associated with enhanced binding to LDL (86). Moreover, the sulfation of GAGs, including both the grade and position of sulfate groups, determines their capacity to bind to LDL (87). chP3R99 monoclonal antibody (mAb), which interacts with sulfated GAGs, efficiently blocks the association of LDL with chondroitin sulfate (CS), thus abrogating LDL oxidation *in vitro*. In line with this, ChP3R99-immunized rabbits showed reduced aortic arch lesions and decreased macrophage infiltration (88). Additionally, chP3R99 has been shown to be atheroprotective in high-fat, high-cholesterol diet-fed ApoE-deficient (−/−) mice due to the generation of anti-GAG antibodies. These antibodies, as a result of an anti-idiotypic cascade, may interfere with the subendothelial retention of LDL via direct binding to GAGs (89). Besides immunization, there are other therapeutic strategies inhibiting LDL-GAG interactions (90), such as ApoB100 peptidomimetics, ligands of PGs, and growth factor inhibitors (91). LDL and PG interaction involves clusters of amino acids in the ApoB100 that bind to the negatively charged sulfate groups on the galactosaminoglycan chain: site A at residues 3,148–3,158 and site B at residues 3,359–3,369 (92). Blocking this interaction by saturating the GAG chain binding sites with small peptide mimetics successfully prevents atherosclerosis in a mouse model (93).

The cytokine named proliferation-inducing ligand (APRIL) is a PG ligand that inhibits the interaction of LDL with heparan-sulfate proteoglycans (HSPGs) (94). Although the physiology of cytokine/HSPG interaction is largely unknown, APRIL confers atheroprotection by limiting LDL retention, intracellular lipid accumulation in macrophages, and formation of necrotic cores in the arterial intima in experimental *in vivo* models. Serum levels of a specific form of human APRIL named non-canonical APRIL (nc-APRIL) are associated with long-term cardiovascular mortality in patients with atherosclerosis, independently of well-established cardiovascular risk factors (95). Imatinib, a platelet-derived growth factor receptor inhibitor mainly used in cancer (96), inhibits sulfate incorporation into PGs, elongation of GAG chain, and reduces xyloside binding to LDL *in vitro* and *in vivo* experimental models (97). Imatinib reduces lipid deposition in the vessel wall without altering circulating lipid levels in high-fat diet-fed ApoE-deficient (−/−) mice (98).

### 3.3 Scavenger and lipoprotein receptors as targets for new immunotherapies against atherosclerosis

Extensive reviews have summarized the connections between innate and adaptive immunity in regulating the immune system and the activation of cytokines and chemokines in the vasculature (99). In this manuscript, we will concentrate on new therapies that raise antibodies blocking the capacity of scavenger receptors to interact with modified lipoproteins.

ROS transform LDL retained in the intima extracellular matrix into oxidized LDL, which is then taken up by macrophages through cholesterol-deregulated scavenger receptors such as CD36 and SRA1, among others, leading to foam cell formation (8).

Immunization with oxidized LDL (oxLDL) has been reported to inhibit atherosclerosis progression in HFD-fed mice (91) and rabbits (100, 101). In addition, specific monoclonal antibodies that recognize oxidation-specific epitopes (OSE) in ApoB100 block oxLDL uptake by macrophages in several experimental *in vivo* models (102–104). Small antibody fragments such as scFv are highly suitable for therapeutic use (105). ASA6 is a new human scFv antibody that interacts with oxLDL and blocks atherosclerosis progression in ApoE−/− mice (106).

In the arterial intima, oxLDL coexists with aggregated LDL, a modified LDL that has a significant impact not only on macrophage function (107, 108), but also on smooth muscle cell (SMC) foam cell formation (109). Low-density lipoprotein receptor-related protein 1 (LRP1) is a key receptor for aggregated LDL-induced foam cell formation (11–13). The inhibition of LRP1 by polyclonal antibodies against the region Gly1127–Cys1140 (P3) located in the CR9 domain of cluster II efficiently blocks aggregated LDL uptake by SMCs (110). Immunotherapy based on the P3 sequence inhibits atherosclerosis in a rabbit model of hypercholesterolemia. Moreover, this immunotherapy, which prevents LRP1 up-regulation, counteracts TNFR1 overexpression involved in VSMC migration into the arterial intima, thus preventing atherosclerosis progression (111).

LRP1 is regulated by the P2Y2 (P2Y purinoreceptor 2) receptor in smooth muscle cells (SMCs) isolated from mice (112). Furthermore, in high-fat diet (HFD)-fed ApoE-deficient (−/−) mice, the P2Y12 receptor increases LRP1-mediated foam cell formation through the upregulation of LRP1 expression (113).

### 3.4 Potential interaction between macrophages and SMC

While the interaction between macrophages and smooth muscle cells (SMCs) in atherosclerotic plaques has been proposed to play a pathological role, primarily through the interchange of cholesterol-rich particles (114, 115), macrophages can also exert a beneficial effect on foam cell formation in SMCs. Specifically, macrophages may contribute to decreasing foam cell formation in SMCs by enhancing the cholesterol efflux capacity of these cells. Exogenous lysosomal acid lipase (LAL) secreted by macrophages appears to be capable of hydrolyzing cholesteryl esters accumulated in the lysosomes of foam SMCs, thereby promoting proper cholesterol efflux (116).

### 3.5 High density lipoproteins (HDL), and reverse cholesterol transport (RCT) activators

High-density lipoproteins (HDLs) play a crucial role in removing excess cholesterol from peripheral tissues and transporting it to the liver for excretion or conversion into bile acids/salts, a process known as reverse cholesterol transport (RCT) (117).

The promotion of free cholesterol (FC) efflux from cholesterol-loaded foam cells situated in the arterial wall is the initial stage of reverse cholesterol transport (RCT). This process is recognized as one of the primary mechanisms responsible for the atheroprotective effects mediated by HDL.

In cholesterol-loaded mouse peritoneal macrophages incubated with diluted human serum, approximately two-thirds of cholesterol efflux occurs through ABCA1 active pathways (118). Moreover, macrophages have been reported to transfer cholesterol to adjacent vascular smooth muscle cells (VSMCs) even in the absence of serum or HDL (119). Interestingly, reverse cholesterol transport (RCT) has been found to be defective in SMCs (120). Both the expression of ATP-binding cassette transporter A1 (ABCA1) and its binding to apolipoprotein A-I (ApoA1) of high-density lipoproteins (HDLs) are reduced in human SMCs, with this impairment appearing to be more pronounced in the advanced stages of atherosclerosis (121). Consistent with these findings, CD45-negative cells (VSMC-derived) from ApoE-deficient (−/−) mice also showed reduced ABCA1 expression and decreased cholesterol efflux to ApoA1 compared to leukocyte-derived foam cells (CD45 positive) (10). However, other studies have reported higher ABCA1 expression in cholesterol-loaded murine SMC (14, 122). Whether SMCs loaded with methyl-β-cyclodextrins-cholesterol reflect mouse SMCs in atherosclerotic lesions is still a matter of debate (121).

Beyond the active efflux through ABCA1 and ABCG1, a recent study showed that HDL-mediated cholesterol efflux is maintained in ABCG1-deficient foam SMCs through the upregulation of SR-BI. This suggests that SR-BI may play a compensatory role in regulating HDL-mediated cholesterol efflux when ABCG1-dependent cholesterol efflux is impaired (123). Lecithin cholesterol acyltransferase (LCAT), a rate-limiting step in reverse cholesterol transport (RCT), plays a crucial role in maintaining free cholesterol (FC) homeostasis between peripheral tissues and HDL. By loading cholesteryl esters (CE) into the core of HDL, LCAT progressively increases particle size, promoting the formation of mature and functional HDL particles (124). New therapeutic approaches based on recombinant human LCAT (rhLCAT), currently in phase II clinical trials (https://www.clinicaltrials.gov/study/NCT04737720), have shown promising results. Preclinical studies indicate that augmenting LCAT levels stimulates reverse cholesterol transport (RCT) and reduces atherosclerosis (125). In familial LCAT deficiency (FLD) patients, intravenous administration of ACP-501, the recombinant human LCAT, improved the abnormal distribution of HDL subfractions and decreased TG levels after meals (126).

## 4 Summary

**Figure 1:**
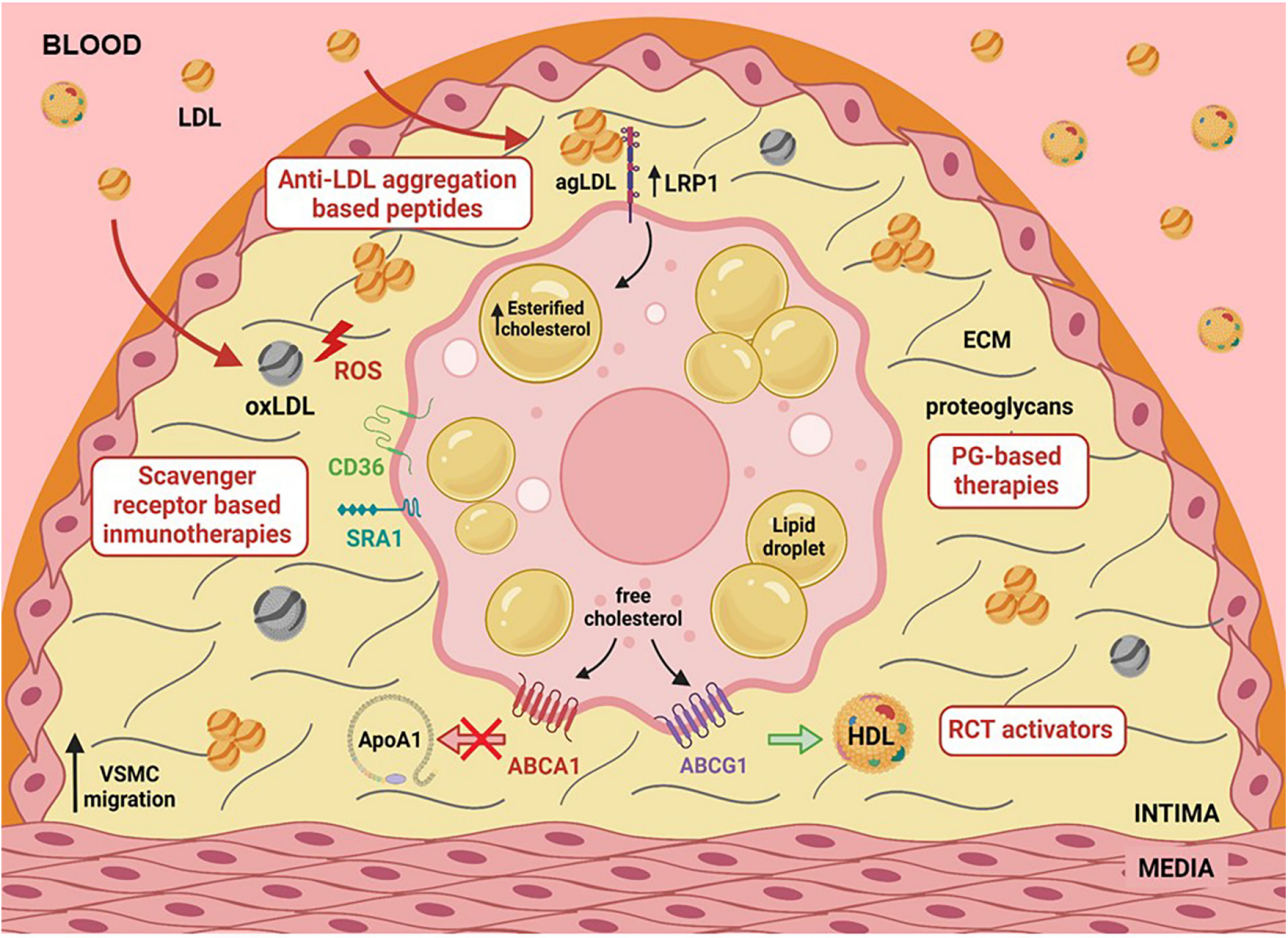
Graphical abstract illustrating various factors such as hypercholesterolemia, hypoxia, and hypertension that upregulate LRP1, a primary lipoprotein receptor involved in human foam SMCs. LRP1 plays a central role in the deregulated uptake of cholesteryl esters (CE) from CE-enriched lipoproteins in SMCs. CE-enriched lipoproteins, predominantly in the form of aggregated LDL, are generated in the extracellular matrix of the arterial intima, interacting with the proteoglycans. The activity of key receptors involved in reverse cholesterol transport (RCT), such as ABCA1 and ABCG1, has been reported to be deficient in foam SMCs. This combination of deregulated CE-enriched lipoprotein uptake and reduced free cholesterol export significantly contributes to the high percentage of foam SMCs in human atherosclerotic plaques.

In summary, the abundant presence of vascular smooth muscle cells (VSMCs) in atherosclerosis was previously overlooked due to their plasticity and synthetic phenotype. The mechanisms regulating cholesterol influx and efflux in VSMC-derived foam cells are still not fully understood. However, targeting SMC foam cells, alongside traditional leukocyte foam cells, will be crucial for effectively treating cardiovascular disease (CVD), as depicted in Figure 1. A proper crosstalk between macrophages and VSMCs could be vital to inhibit the progression of atherosclerosis.
