# Correction: Mechanisms modulating foam cell formation in the arterial intima: exploring new therapeutic opportunities in atherosclerosis

**DOI:** 10.3389/fcvm.2026.1808883

**Published:** 2026-04-20

**Authors:** M. T. La Chica Lhoëst, A. Martinez, L. Claudi, E. Garcia, A. Benitez-Amaro, A. Polishchuk, J. Piñero, D. Vilades, J. M. Guerra, F. Sanz, N. Rotllan, J. C. Escolà-Gil, V. Llorente-Cortés

**Affiliations:** 1 Department of Experimental Pathology, Institute of Biomedical Research of Barcelona (IIBB)-Spanish National Research Council (CSIC), Barcelona, Spain; 2 Department of Cardiovascular, Institut de Recerca Sant Pau (IR SANT PAU), Barcelona, Spain; 3 Research Programme on Biomedical Informatics (GRIB), Department of Experimental and Health Sciences (DCEXS), Hospital del Mar Medical Research Institute (IMIM), Universitat Pompeu Fabra (UPF), Barcelona, Spain; 4 Department of Cardiology, Hospital de la Santa Creu I Sant Pau, Biomedical Research Institute Sant Pau (IIB-SANTPAU), Universitat Autonoma de Barcelona, Barcelona, Spain; 5 Department of Cardiovascular, CIBERCV, Institute of Health Carlos III, Madrid, Spain; 6 Department of Cardiovascular, CIBERDEM, Institute of Health Carlos III, Madrid, Spain

**Keywords:** apolipoproteins (ApoB100, apoA1, apoC1, apoJ, apoE), foam-like SMC, atherosclerosis, cardiovascular diseases, peptidomimetics, lipoproteins, transcription factors, reverse cholesterol transport

In the published article, an incorrect Funding statement was provided. In the first sentence, the wording “co-financed with ERDFs” for grant FIS PI21/01523 (to VLL-C) was incorrect and should read “co-funded by the European Union.” The corrected Funding statement appears below:

“The economic support to develop this project was received from FIS PI21/01523 (to VLL-C) from the Instituto de Salud Carlos III (ISCIII) and co-funded by the European Union, and Fundación BBVA Ayudas a equipos de investigación 2019. Aleyda Benitez Amaro (ABA) is a postdoctoral fellow (FI19/00205) that was granted by the Programme Contratos predoctorales de formación de investigación en salud_from the Instituto de Salud Carlos III (ISCIII) and co-financed with ERDFs. The RIFS methodology required to analyze mitochondrial respiration in frozen cardiac samples was developed thanks to the AYUDAS PARA LA MOVILIDAD DE PERSONAL INVESTIGADOR CONTRATADO EN EL MARCO DE LA AES (M-AES) del ISCIII granted by ABA (MV21/00060). Our group is part of CIBER Enfermedades Cardiovasculares (CIBERCV; CB16/11/00276 to VLl-C), and CIBER de Diabetes y Enfermedades Metabólicas Asociadas (CIBERDEM; CB07/08/016 to JC-EG and NR), projects run by the Instituto de Salud Carlos III. Our group also participates in Redes de investigación (Enfermedades Metabóloicas y Cáncer RED2018- 102799-T), a project run by MINECO. We belong to a group recognized by Generalitat de Catalunya (2021 SGR 00834). This work was also funded by Ministerio de Ciencia, Innovación y Universidades (PID2022-137186OB-100), as well as from the Agencia Estatal de Investigación (AEI/10.13039/501100011033) within the Subprograma Ramón y Cajal (RYC-201722879) to NR. The IR SANT PAU is a centre of CERCA Programme/Generalitat de Catalunya”.

The original version of this article has been updated.

